# Utilization of Electrocardiographic P-wave Duration for AV Interval Optimization in Dual-Chamber Pacemakers

**Published:** 2010-09-05

**Authors:** Dan Sorajja, Mayurkumar D Bhakta, Luis RP Scott, Gregory T Altemose, Komandoor Srivathsan

**Affiliations:** Division of Cardiovascular Diseases, Mayo Clinic Arizona, 5777 E Mayo Blvd, Phoenix, AZ 85054, USA

**Keywords:** Pacing, Echocardiography, AV interval, Optimization, Electrocardiogram

## Abstract

**Background:**

Empiric programming of the atrio-ventricular (AV) delay is commonly performed during pacemaker implantation. Transmitral flow assessment by Doppler echocardiography can be used to find the optimal AV delay by Ritter's method, but this cannot easily be performed during pacemaker implantation. We sought to determine a non-invasive surrogate for this assessment. Since electrocardiographic P-wave duration estimates atrial activation time, we hypothesized this measurement may provide a more appropriate basis for programming AV intervals.

**Methods:**

A total of 19 patients were examined at the time of dual chamber pacemaker implantation, 13 (68%) being male with a mean age of 77. Each patient had the optimal AV interval determined by Ritter's method.  The P-wave duration was measured independently on electrocardiograms using MUSE® Cardiology Information System (version 7.1.1). The relationship between P-wave duration and the optimal AV interval was analyzed.

**Results:**

The P-wave duration and optimal AV delay were related by a correlation coefficient of 0.815 and a correction factor of 1.26. The mean BMI was 27. The presence of hypertension, atrial fibrillation, and valvular heart disease was 13 (68%), 3 (16%), and 2 (11%) respectively. Mean echocardiographic parameters included an ejection fraction of 58%, left atrial index of 32 ml/m^2^, and diastolic dysfunction grade 1 (out of 4).

**Conclusions:**

In patients with dual chamber pacemakers in AV sequentially paced mode and normal EF, electrocardiographic P-wave duration correlates to the optimal AV delay by Ritter's method by a factor of 1.26.

## Introduction

The implantation of a dual chamber pacemaker may serve as an alternate timing mechanism for the conduction system of the heart. These devices try to mimic intrinsic automaticity, and they also allow adjustment of the timing of the atrio-ventricular excitation sequence.  As such, the effects of DDD programming pacing affects right heart hemodynamics, and due to interventricular dependence impacts left heart hemodynamics [1,2]. To optimize left ventricular filling in these patients, the AV delay must be programmed short enough to avoid premature mitral valve closure with mitral regurgitation, and long enough to avoid left atrial cannon waves [3,4]. Techniques such as impedance cardiography [4,5] and analysis of aortic valve hemodynamics [6] have utility in assisting the programmer to define the optimum timing interval.  In patients with complete heart block and DDD pacemakers, the Ritter method is one of several methods used to optimize the AV delay by synchronizing left atrial and ventricular contractions to allow for maximal cardiac output [7].  The Ritter method has also been applied to patients with preserved and reduced ejection fractions [3], and for cardiac resynchronization therapy [8,9].

The P-wave duration has been shown to correlate to interatrial conduction time with the initial and terminal portions of the P wave corresponding to the right and left atrial activation respectively [10-13]. However, interatrial conduction times vary significantly between individuals, thereby influencing the optimal AV interval [12,14,15]. Atrioventricular conduction is also known to be variable, manifested by beat-to-beat PR interval variability in patients with and without coronary artery disease [16]. In addition, the influence of the autonomic nervous system on atrioventricular conduction is well described [17,18]. The size of the person's body has minimal effect on the PR interval [19]. These interatrial and atrioventricular conduction variabilities have implications for the optimal timing of DDD pacing. Allowing for a fixed electromechanical coupling interval, P-wave duration and optimal AV interval is likely to have a reproducible relationship. The goal of our study was to evaluate if electrocardiographic P-wave duration would correlate with optimal AV delay as calculated by Ritter's method.

## Materials and Methods

Nineteen patients with dual chamber pacemakers were included in the study.  All patients had prolonged PR interval (> 200 ms) or high degree AV block.  No fusion or pseudofusion was present during measurements taken at rest.  Baseline characteristics of the study group were collected.  To verify placement of the atrial lead in the right atrial appendage and the right ventricular lead in the right ventricular apex, the operative fluoroscopy and post-procedure chest x-ray films were reviewed.

Echocardiograms were obtained in the left lateral decubitus position using a Vivid-I Cardiovascular Ultrasound (GE Healthcare, Milwaukee, Wisconsin).  The apical 4-chamber and 2-chamber views were used from end-diastole and end-systole for calculation of ejection fraction and left atrial volume.  The left atrial index was determined by dividing the left atrial volume by the body mass index.  Grading of diastolic dysfunction was carried out by previously published criteria [20].

**Figure 1:**
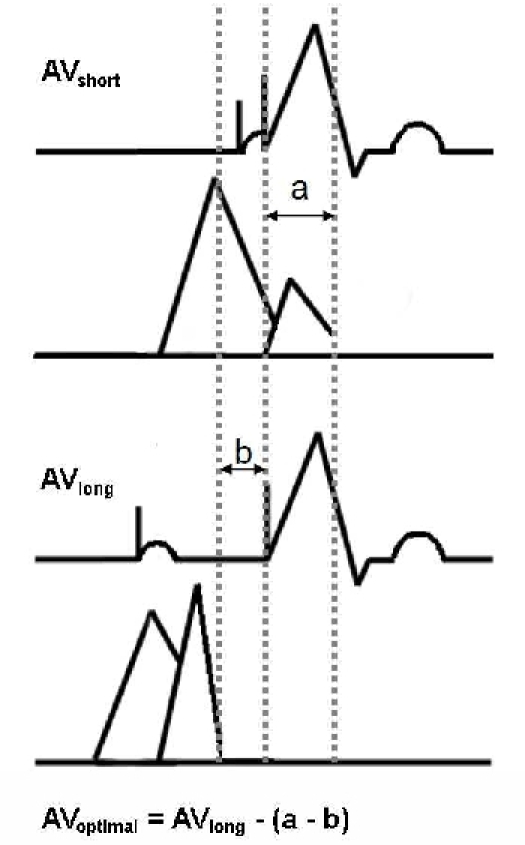
Calculation of Optimal Atrioventricular (AV) Delay by Mitral Inflow Pattern. While using Doppler echocardiography, the pacemaker is programmed to a non-physiologically short AV delay.  The value "a" encompasses the time interval between ventricular pacing artifact to the end of the A wave in the mitral flow.  The pacemaker is then programmed to a long AV delay.  The value "b" includes the time interval between the end of the A wave and the ventricular pacing artifact.  The optimal AV interval is then calculated by subtracting "b" from "a" and subtracting this calculated value from the long AV delay value.

The optimal AV delay was calculated using an AV paced rhythm with the Ritter method, based on pulsed wave Doppler echocardiography of the transmitral blood flow (Figure 1) [7]. This method requires several steps. First, the pacemaker is programmed to a non-physiologically short AV delay, causing mitral valve closure to occur with the onset of left ventricular contraction. This value "a" is the longest interval, encompassing the ventricular pacing artifact to the end of the A wave in the mitral flow velocity ("a" is the electromechanical delay between right ventricular stimulation and the beginning of the left ventricular systole). Next, the pacemaker is programmed to a long AV delay to determine "b". This value "b" includes the time interval between the ventricular pacing artifact and the end of the A wave. By correcting the long AV delay by the values "a" and "b", the timing of ventricular systole can be optimized to allow for maximum diastolic ventricular filling.  The calculation of the optimal AV interval (AVopt = AVlong - [a - b]) thereby prevents the occurrence of left atrial cannon waves and diastolic mitral regurgitation.

For P-wave duration, the ECG obtained prior to device implantation was used for measurements. A 12-lead standard surface electrocardiogram (10 mm/mV, 25 mm/s) was obtained in the supine resting position using a computer-based ECG system (MUSE® Cardiology Information System, Version 7.1.1, General Electric, Milwaukee, Wisconsin). Subjects were breathing freely during acquisition, but not allowed to speak. The MUSE® program calipers were used to measure the P wave in leads II and V1, and the operator was blinded to echocardiographic findings.  The onset and offset of the P wave were defined as the start of the upward deflection of the P-wave pattern and its return to the isoelectric baseline in lead II [21]. Right atrial abnormality was defined as a P-wave height greater than 2.5 mm in lead II. Left atrial abnormality was defined as P-wave duration greater than 120 ms in lead II or a negative deflection greater than 1 mm of the P wave in lead V1 [22]. The P-wave duration was then plotted against the optimal AV delay and other baseline characteristics and findings.  Regression analysis was performed.

Informed consent was obtained from all study participants, and this study was approved by the Mayo Foundation Institutional Review Board.

## Results

There were 19 patients, 13 male and 6 female, mean age 77 +/- 5 years.    The average BMI was 27.  A history of atrial fibrillation was present in 3 (16%) patients.  Ten patients had left atrial enlargement on echocardiography, with the mean left atrial index being 32 ml/m2.  On electrocardiogram, right and left atrial abnormalities were present in 0 (0%) and 12 (63%) patients respectively.  Other baseline characteristics are summarized in Table 1.  A summary of pacemakers used is included in Table 2.

The average P-wave duration on electrocardiogram was 113 ± 19 ms, ranging from 88 to 140 ms.  For patients with a history of atrial fibrillation, the P-wave duration was 95 ± 12 ms. Patients with electrocardiographic left atrial abnormality had a mean P-wave duration of 120 ± 19 ms.  In patients with left atrial enlargement on echocardiogram, the mean P-wave duration was 119 ± 20 ms.  Overall, the mean heart rate and PR interval were 53 ± 13 bpm and 201 ± 34 ms respectively.  The optimal AV delay calculated by Ritter's method was 142 ± 40 ms for all patients. Other optimal AV delay calculated for the presence of any interventricular conduction delay is summarized in Table 3.

**Figure 2:**
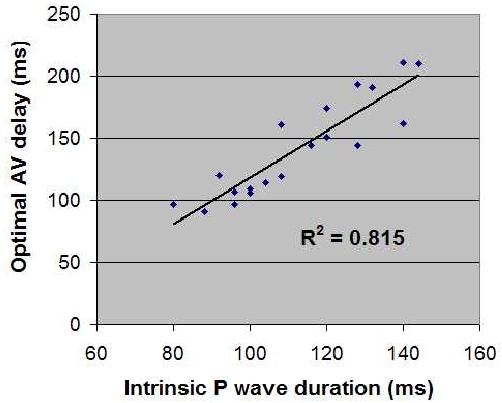
Correlation of P Wave Duration to Optimal Atrioventricular (AV) Delay. The plot was constructed using P-wave durations and optimal AV delay as determined by Ritter’s method.  The correlation coefficient is high, indicating that optimal AV delay can be predicted from P-wave duration in patients with dual-chamber pacemakers.

The correlation coefficient for the P-wave duration and optimal AV delay was 0.815 (Figure 2).  Dividing the mean optimal AV delay by the mean P wave duration gives a ratio of 1.26 ± 0.24 ms for the entire cohort. In patients with LBBB and nonspecific interventricular conduction delay, the ratio was similar, 1.11 to 1.13. In patients with RBBB and those without any interventricular conduction delay, the ratio was similar 1.31 (Table 3). The optimal AV delay did not correlate to the left atrial index (R^2^ = 0.21), electrocardiographic heart rate (R^2^ = 0.07), or electrocardiographic PR interval (R^2^ = 0.26).

## Discussion

For patients undergoing dual chamber pacemaker implantation, measurement of the electrocardiographic P-wave duration correlates by a ratio of 1.26 to the optimal AV delay by Ritter's method when pacemaker is in AV sequential pacing mode.  This ratio gives clinicians a useful tool to program the AV delay based on the electrocardiographic P wave duration.

By adding one-fourth of the P-wave duration to its baseline measurement, device implanters and programmers can calculate the likely optimal AV delay during AV pacing within a reasonable degree of certainty.  This calculation provides an alternative to the use of empiric device settings that may not be hemodynamically suitable for individual patients.  This calculation is unlikely to replace cardiac output optimization methods such as echocardiography in dual-chamber pacemaker patients, but implementation of this calculation could improve cardiac hemodynamics in patients who have yet to undergo such optimization methods, which requires time, availability, and a trained echocardiographer to perform [23]. While many patients will likely not suffer any serious consequences with programming of the AV delay slightly shorter or longer than the optimal AV delay, the improvement by this simple optimization method could potentially improve hemodynamics in many of them.  For patients with dual chamber pacemakers, the optimal AV interval can lead to significant improvement in mechanical atrio-ventricular synchrony and quality of life [4,24,25]. The late diastolic mitral regurgitation, seen with first-degree heart block and complete heart block, can be reduced or eliminated with AV delay optimization leading to improved stroke volume, which may benefit heart failure patients in particular [3,26]. For these reasons among others, empiric programming of the AV interval is not recommended [27,28]. However, the benefit of AV synchrony may not be apparent if the right ventricle is frequently paced to maintain this synchrony. With high burdens of right ventricular pacing, interventricular dyssynchrony may develop and reduce cardiac output and function, particularly those patients with congestive heart failure [29,30]. However, the vast majority of patients will not have their left ventricular ejection fraction compromised by frequent right ventricular pacing [31], The utility of AV interval programming based on P-wave duration as suggested in this study is beneficial for patients with AV conduction abnormalities who are in AV sequential pacing mode.

Acceptable intraobserver and interobserver measurement of P wave duration has been shown in a number of studies [32-34].  In addition, the computerized on-screen measurement of P-wave duration (including the MUSE system as used in this study) has been reported to have the lowest intraobserver and interobserver variability with an error of 3 ± 2.9%, superior in comparison to both manual measurement of P-wave duration in electrocardiograms magnified 200% and high resolution digitizing board with on-board measurement of P-wave duration [21].

Although electrocardiographic P-wave duration is known to correlate to interatrial conduction times in sinus and right atrial pacing modes [14], certain patient groups may benefit from a shorter or longer AV delay in relation to the P wave duration depending on factors such as lead placement location, sinus versus paced rhythm, and interventricular delay. All of our patients had the right atrial lead placed in the appendage, which is a known contributor to interatrial conduction delay. Patients with the right atrial leads placed septally would likely need shorter AV delays programmed [13]. Shorter calculated AV delays may also be necessary if the terminal component of the P wave is felt to represent pulmonary vein activation, and not atrial activation  [35]. Inclusion of the terminal portion of the P wave in these cases would lead to overestimation of the optimal AV delay.  Patients who pace the majority of time in an atrial tracking mode may require a shorter AV interval, since interatrial conduction times prolong with right atrial pacing when compared to sinus rhythm.  In one study of patients with electrocardiographic P-wave duration of less than 110 ms and greater than 110 ms, the average interatrial conduction time lengthened 26 and 27 ms on average respectively with right atrial pacing [14]. However, detection of a P wave by a pacemaker in an atrial-sensing mode takes an average of 30 ms [2]. The lengthening of the interatrial conduction time with atrial pacing may offset the delay in detection of a P wave during an atrial-sensing mode but these intervals substantially differ from patient to patient and this difference has to be taken in consideration in programming the AV delay.  Also, the interatrial conduction time is nearly constant at all atrial paced rates between 80 to 160 bpm, varying only 7 ms, so further adjustment to the optimal AV delay based on the pacing rate may not be necessary [14].

Patients with a history of paroxysmal atrial fibrillation may benefit from a longer correction factor to calculate the optimal AV delay, since these patients typically have an increased P wave duration when in sinus rhythm although this was not seen in our limited patient cohort [21,32,36].

Limitations of this study include the small study population in a tertiary medical center. With the limited number of patients, the current study could be considered a pilot study and application to larger populations needs further investigation. Although some referral-related bias may be present, our patients have characteristics in common with patients that would be seen in a general practice.   Specific measurements of P-wave sensing delay, paced interatrial delay, and interventricular delay were not measured in individual patients.   Optimizing the AV delay for both exercise and increased heart rate was also not performed in our cohort, although rate-adaptive shortening of the AV delay is of known benefit in patients with DDDR pacemakers with normal ejection fractions [28]. No long term follow-up was undertaken after programming of the device to the optimal AV delay.  While our study correlates the P-wave duration to the optimal AV delay, no specific analysis on cardiac output or hemodynamics was performed.  Analysis of the effect of the optimal AV delay on cardiac hemodynamics, quality of life, and other parameters would require another study design.  The reproducibility of our findings may also depend on the availability of computerized on-screen measurement of ECG parameters, including the P wave, as well as having P waves large and distinct enough to measure in patients.  In patients with no visible P waves, this data is not   applicable.

Our study shows the P-wave duration correlates to the optimal AV delay as calculated by Ritter's method by a factor of 1.26.  Using this ratio, clinicians can determine an individual's optimal AV delay based on a patient's own electromechanical activation. Our simple calculation gives clinicians a useful tool that should benefit patients beyond empiric device settings, which may not be hemodynamically suitable for patients.  With the limited number of patients, the current study could be considered a pilot study and application to larger populations needs further investigation.

## Figures and Tables

**Table 1:**
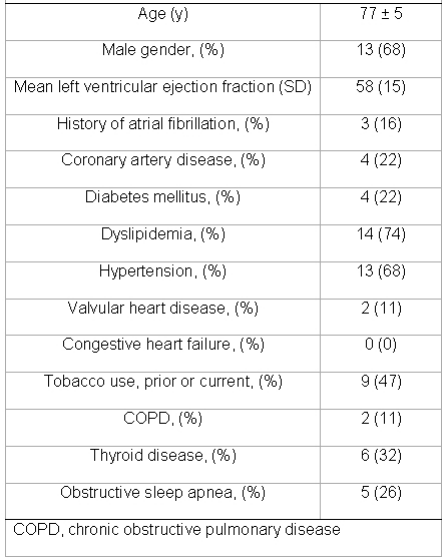
Clinical characteristics of the patients

**Table 2:**
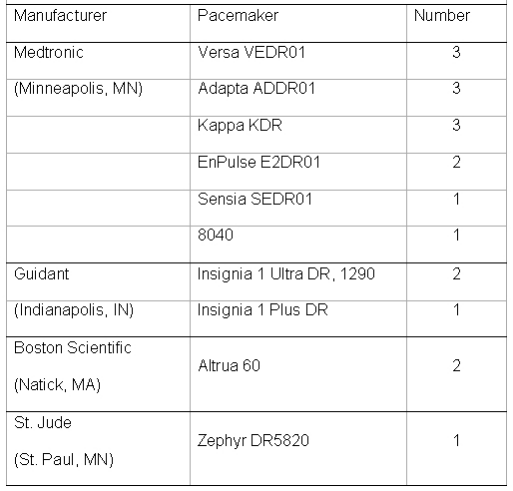
Pacemaker Models, Manufacturers, and Number

**Table 3:**
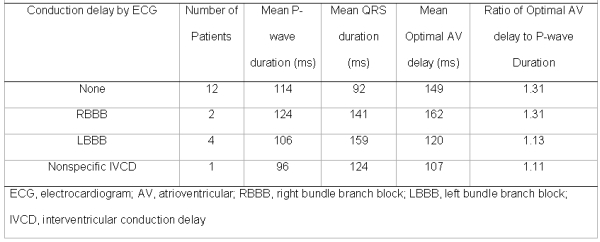
Relation of ECG characteristics to optimal AV delay
